# Tuberculosis Preceding Lung Cancer: A Contemporary Meta-Analysis Revealing a Critical Gap in Post-2020 Evidence

**DOI:** 10.3390/cancers18071097

**Published:** 2026-03-28

**Authors:** Cristina Cioti, Irina Tica, Miruna Cristian Gherase, Gabriela Fricatel, Oana Cristina Arghir

**Affiliations:** 1 Internal Medicine Department, “Sf. Apostol Andrei” Emergency County Hospital, 145 Tomis Blvd., 900591 Constanta, Romania; irinatica73@yahoo.com; 2 School of Medicine, “Ovidius” University of Constanta, 1 University Street, 900470 Constanta, Romania; miruna.cristian@365.univ-ovidius.ro (M.C.G.); fricatelgabriela@yahoo.com (G.F.); arghir_oana@yahoo.com (O.C.A.); 3 Center for Research and Development of the Morphological and Genetic Studies of Malignant Pathology—CEDMOG, “Ovidius” University of Constanța, Aleea Universității nr. 1, Campus-Corp A, etaj 1, 900470 Constanta, Romania; 4 Oncology Department, “Sf. Apostol Andrei” Emergency County Hospital, 145 Tomis Blvd., 900591 Constanta, Romania; 5 Clinical Pneumology Hospital of Constanta, 40 Sentinelei Str., 900002 Constanta, Romania

**Keywords:** tuberculosis, lung cancer, carcinogenesis, chronic inflammation, meta-analysis, immune dysregulation, pulmonary fibrosis, epidemiology

## Abstract

Background: Tuberculosis (TB) has long been suspected to contribute to lung carcinogenesis through chronic inflammation and immune dysregulation. However, contemporary controlled evidence quantifying this association remains limited. We aimed to systematically evaluate the relationship between prior TB and subsequent lung malignancy, using recent observational studies and complementary case reports. Methods: A systematic review and random-effects meta-analysis were conducted, including controlled cohort and case–control studies published from 2020 onward. Adjusted effect estimates were converted to the logarithmic scale for pooling. Heterogeneity and small-study effects were assessed using standard meta-analytic techniques. Additionally, published case reports were descriptively analyzed to explore clinicopathological patterns. Results: Across eligible studies, prior TB was consistently associated with an increased risk of subsequent lung cancer (LC). The pooled estimate demonstrated a statistically significant positive association, despite moderate heterogeneity. Larger nationwide cohorts contributed greater statistical weight, while smaller studies showed wider variability. Case reports revealed heterogeneous temporal patterns, including long-latency scar-associated carcinoma and concurrent inflammatory–malignant presentations. Conclusions: Contemporary controlled evidence supports an association between prior tuberculosis and increased risk of subsequent lung malignancy. However, despite strong biological plausibility and the abundant literature on cancer-associated tuberculosis, modern longitudinal studies specifically evaluating tuberculosis as a preceding independent risk factor remain limited. The small number of eligible post-2020 investigations identified in this meta-analysis highlights a significant contemporary research gap and underlines the need for well-designed prospective studies to clarify causality and guide surveillance strategies in TB-exposed populations.

## Simple Summary

The hypothesis that tuberculosis predisposes to subsequent lung cancer has biological plausibility but remains insufficiently addressed in contemporary controlled research. Notably, most recent investigations emphasize tuberculosis occurring during cancer treatment, rather than tuberculosis as a preceding and independent risk factor. We therefore conducted a meta-analysis restricted to post-2020 population-based studies and case reports to evaluate this temporal association, using adjusted risk estimates. We found a statistically significant increase in lung cancer risk among individuals with prior tuberculosis. Importantly, the limited number of eligible modern studies highlights a substantial gap in the high-quality longitudinal evidence.

## 1. Introduction

**Figure 1:**
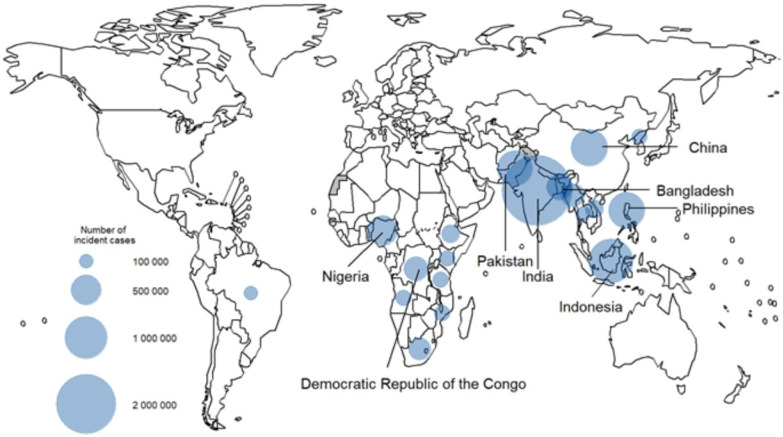
Estimated number of incident TB cases in 2023, for countries with at least 100,000 incident cases [3].

Tuberculosis and lung cancer represent two major global health challenges whose coexistence has increasingly attracted clinical and scientific attention [1]. The incidence of lung cancer among patients with TB has been reported to be two to four times higher than that observed in the general population [2]. Figure 1 shows the estimated number of TB incident cases worldwide.

Epidemiological data further suggest that TB patients may exhibit a 1.5–11-fold higher incidence of lung cancer compared with non-TB populations, and that a measurable proportion of patients with either condition develop comorbidity [4]. Large cohort analyses have demonstrated a markedly elevated risk of lung cancer shortly after TB diagnosis, with hazard ratios exceeding 12 within the first year and remaining significantly increased during subsequent follow-up [5].

**Figure 2:**
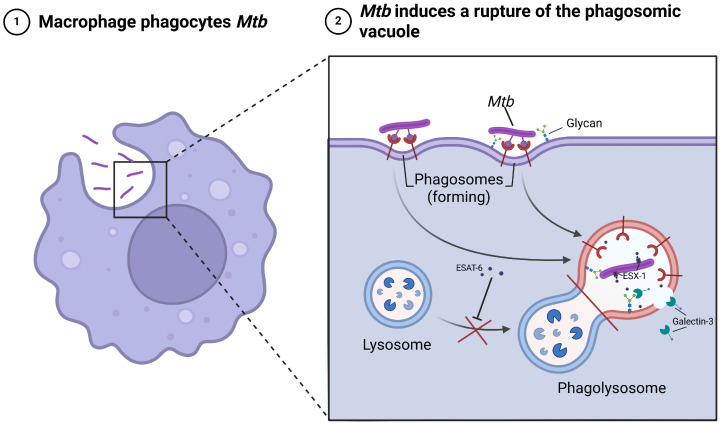
Mechanism of *Mtb* phagosomal escape and intracellular persistence in macrophages. Created in BioRender. Cristina, C. (2026) https://BioRender.com/4jugpix (accessed on 20 March 2026) [8].

Mechanistically, chronic pulmonary inflammation and fibrosis induced by TB are thought to contribute to carcinogenesis [6]. Figure 2 illustrates the process by which macrophages phagocytose *Mycobacterium tuberculosis* (Mtb), followed by ESX-1-mediated phagosomal membrane disruption, enabling bacterial escape and persistence within the host cell [7].

The inhibition of effective phagolysosomal fusion and the subsequent activation of chronic inflammatory pathways promote sustained immune stimulation and tissue remodeling [9]. Persistent Mtb infection may induce prolonged production of reactive oxygen and nitrogen species, DNA damage, and cytokine-driven microenvironmental alterations [10]. Such chronic inflammatory signaling and immune dysregulation are biologically relevant mechanisms that may contribute to fibrosis, the epithelial–mesenchymal transition and, ultimately, carcinogenesis, thereby providing a mechanistic link between prior tuberculosis and the development of lung malignancy [11].

**Figure 3:**
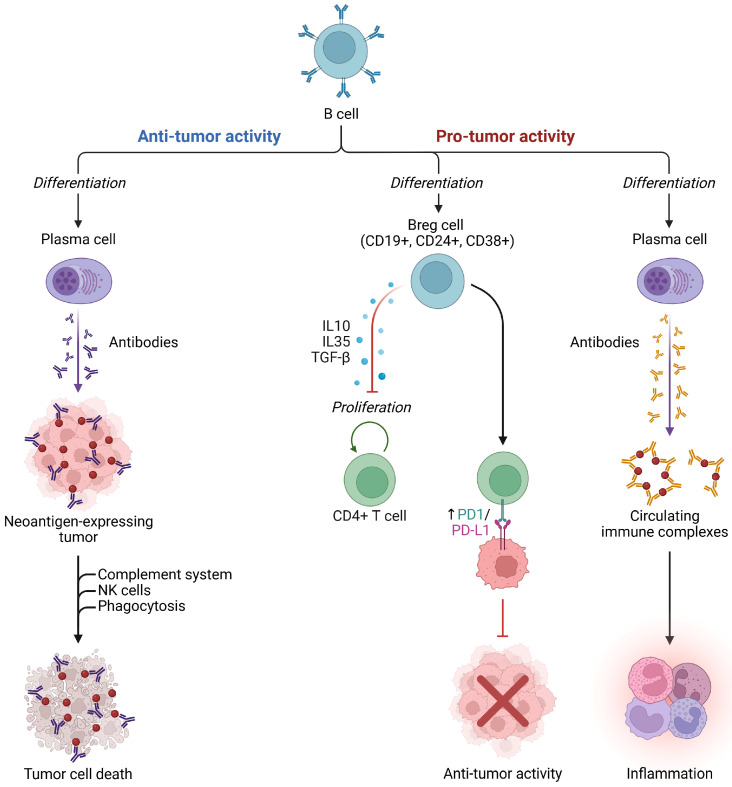
Anti-tumor and pro-tumor activity of B cells in the cancer tumor microenvironment. Created in BioRender. Cristina, C. (2026) https://BioRender.com/6jl21gb (accessed on 20 March 2026) [8].

At the tumor–immune interface (Figure 3), *Mtb* infection has been associated with reduced T-cell infiltration and increased PD-L1 expression in lung adenocarcinoma, suggesting that TB-related immune modulation may enhance tumor aggressiveness [12].

Clinically, the coexistence of TB and lung cancer presents significant diagnostic and therapeutic challenges, due to overlapping radiological and clinical features [13].

Immunomodulatory therapies, particularly PD-1/PD-L1 inhibitors, may further complicate management by potentially triggering TB reactivation [14].

In light of these epidemiological associations, mechanistic insights, and clinical complexities, the present study aims to systematically evaluate the relationship between TB and subsequent lung malignancy by synthesizing cohort-level evidence and case-based data, and to clarify the magnitude, patterns, and potential biological underpinnings of this association [15,16,17].

Notably, although historically, the literature has explored the association between tuberculosis and lung cancer, most contemporary publications focus on cancer-associated tuberculosis or TB reactivation during oncologic therapy. Modern controlled studies specifically evaluating tuberculosis as a preceding and independent risk factor for subsequent lung malignancy remain comparatively limited, particularly in the post-2020 literature.

## 2. Materials and Methods

### 2.1. Study Design and Protocol

This study was conducted as a systematic review and meta-analysis of observational studies evaluating the association between TB and subsequent lung malignancy.

The year 2020 was selected as the starting point for this review to capture the transition into a contemporary oncologic era marked by the widespread adoption of immunotherapy and advancements in epidemiological reporting standards. Nevertheless, to preserve contextual completeness and ensure a balanced interpretation of the evidence, relevant studies published prior to 2020 were also considered within the discussion. This approach enables a structured comparison between historical and recent data, thereby providing a more comprehensive perspective on the evolving understanding of the association between tuberculosis and lung cancer.

Thus, the search was conducted in the PubMed/MEDLINE database to identify eligible studies published from 1 January 2020 to the date of the final search (21 February 2026). The search strategy was developed using Medical Subject Headings (MeSH) in combination with free-text terms [18].

The following MeSH terms were applied: “Tuberculosis” [MeSH], “Lung Neoplasms” [MeSH], “Carcinogenesis” [MeSH], “Inflammation” [MeSH], “Pulmonary Fibrosis” [MeSH], and “Risk Factors” [MeSH]. These terms were combined using Boolean operators, as follows [19]:

(“Tuberculosis” [MeSH] OR tuberculosis) AND (“Lung Neoplasms” [MeSH] OR “lung cancer”) AND (“Risk Factors” [MeSH] OR carcinogenesis OR inflammation).

Filters were applied to restrict results to human studies published in English. Reference lists of included articles were manually screened to identify additional relevant studies.

The use of a single database may have limited the breadth of the search; however, manual reference screening did not identify additional eligible studies, suggesting that the risk of missing relevant data is low.

**Figure 4:**
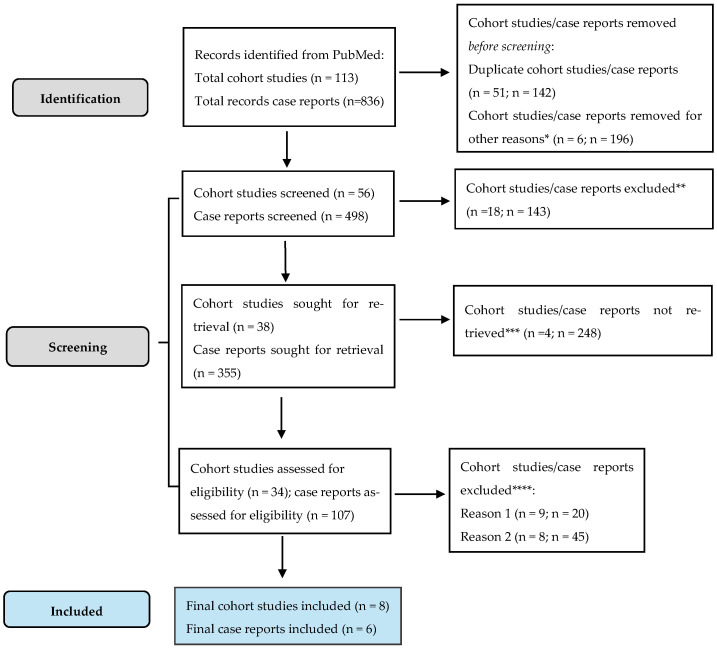
PRISMA flowchart. * Studies are not relevant for the present review; ** non RCT, wrong population; *** unable to find the full text of the study; and **** Reason 1—study on animals/Reason 2—wrong setting/Reason 3—research question not relevant.

This meta-analysis was prospectively registered in the PROSPERO international database (CRD420251050743) [20]. The registered title was subsequently refined for clarity and terminological precision during manuscript preparation. No modifications were made to the research question, eligibility criteria, outcomes, or methodological approach. The review was conducted in accordance with the predefined protocol and reported following the PRISMA guidelines (Figure 4) [21].

The full breakdown of PRISMA selection is included in Supplementary Material S1.

### 2.2. Eligibility Criteria

Studies were eligible if they:

- Evaluated the association between TB and subsequent lung cancer.
- Reported adjusted risk estimates (HR, sub-HR, SIR, or OR) with 95% confidence intervals.
- Included cohort or case–control designs.
- Provided sufficient data for effect size conversion.

Exclusion criteria:

- Non-human studies.
- Reviews or editorials.
- Studies without extractable effect estimates.
- Overlapping populations without distinct analyses.

Case reports were included separately for descriptive analysis but were not pooled in quantitative synthesis.

### 2.3. Data Extraction

For each study, the following variables were extracted: study design, country, data source, sample size (total, TB, non-TB), mean or median age, sex distribution, follow-up duration, lag period application, TB definition, lung cancer definition, adjusted effect estimates with 95% CI, and covariates included in multivariable models [22].

For meta-analysis, effect estimates were transformed to the log scale using the following [23]:

log (HR)

Standard errors (SE) were calculated from confidence intervals using the following [24]:

$$SE=\frac{log\;(UpperCI)-log\;(LowerCI)}{2\times 1.96}$$

### 2.4. Effect Size Harmonization

Hazard ratios (HR), sub-HR, and standardized incidence ratios (SIR) were treated as comparable relative risk measures for pooling. Odds ratios (OR) from case–control studies were not included in the main HR pooling, but were reported descriptively [25,26,27].

All estimates were converted to log(HR) to stabilize the variance and ensure normal distribution assumptions [28].

### 2.5. Statistical Analysis

A random-effects meta-analysis model was applied to account for between-study heterogeneity. The pooled estimate was calculated using inverse-variance weighting [29]:

$$Weight_{i}=\frac{1}{SE_{i}^{2}+\tau^{2}}$$

where τ^2^ represents between-study variance. The heterogeneity was assessed using Cochran’s Q test, I^2^ statistic, Tau^2^. Statistical significance was defined as *p* < 0.05.

### 2.6. Meta-Regression and Bubble Plot Analysis

A meta-regression analysis was performed under a random-effects framework to explore the influence of study-level moderators on effect size. Bubble plots were generated, with the bubble size being proportional to the study weight.

### 2.7. Case Report Synthesis

Published case reports were analyzed descriptively. Variables included age and sex, TB type, interval between TB and cancer, TB status at diagnosis, histology, tumor stage, treatment, clinical outcome and relationship pattern. Categorical coding was applied to standardize variables for visualization and network analysis, and the complete coding framework, together with the structured dataset, is presented in Supplementary Material S2 (Table S1).

### 2.8. Software

All statistical analyses were performed using IBM SPSS Statistics version 29 (Meta-Analysis module) [30]. Forest plots, funnel plots, bubble plots, and additional graphical representations were generated within the SPSS environment.

## 3. Results

### 3.1. Analysis of Study Cohorts

#### Population Characteristics

A total of eight studies were included in the qualitative synthesis (Table 1), comprising seven population-based cohort studies and one hospital-based case–control study conducted in East Asia (South Korea, Taiwan, and China) [31,32,33,34,35,36,37,38]. Most investigations were nationwide retrospective cohorts utilizing large administrative or registry-linked datasets. South Korean studies were primarily based on the National Health Insurance Service (NHIS) or its derivative cohorts [31,32,34], the KNHANES database linked to the national cancer registry [33], or regional tuberculosis registry data linked to NHIS claims [35]. Taiwanese studies used the National Health Insurance Research Database (NHIRD) [36,37], while the Chinese investigation was a hospital-based case–control study with pathologically confirmed lung cancer cases [38].

Across the cohort studies with internal comparators, the sample sizes ranged from 13,165 participants in a COPD subgroup analysis [34] to 229,225 individuals in the Taiwanese nationwide cohort [36]. Moon et al. [32] included 150,934 participants with balanced TB and non-TB groups, whereas An et al. [31] analyzed 22,656 individuals from a nationally representative sample cohort. Hong et al. [35] evaluated 35,140 tuberculosis patients without an internal control group, reporting standardized incidence ratios relative to the general population. The Chinese case–control study included 1776 TB patients and 30,763 controls [38].

The baseline demographic characteristics were broadly consistent across studies. Most cohorts were male-predominant, with male proportions ranging from 52% in the COPD subgroup cohort [34] to 72.4% in the Taiwanese cohort evaluating secondary lung cancer [37]. The KNHANES-linked study by Oh et al. [33] was the only cohort in which males constituted less than half of the study population (42.5%). The mean or median age across studies ranged from 55 years in the Chinese case–control study [38] to 67 years in the Taiwanese secondary lung cancer cohort [37], indicating that most participants were middle-aged or elderly.

The follow-up duration varied considerably. The mean follow-up ranged from 3.9 years in the KNHANES-linked cohort [33] to 8.0 years in the registry-linked TB cohort [35], while Ho et al. [37] reported follow-up extending up to 16 years. Several studies incorporated lag-period exclusions to reduce potential reverse causality, including 6-month exclusions [33], 1-year exclusions with additional sensitivity analyses [32,35], or sensitivity analyses excluding 6–12 months after TB diagnosis [34]. In contrast, An et al. [31] and Ho et al. [37] did not apply formal lag exclusions, although An et al. stratified the risk by time since TB diagnosis.

Tuberculosis definitions were predominantly based on ICD coding algorithms (ICD-10 A15–A19 or ICD-9-CM 010–018), which are often combined with anti-tuberculosis prescription criteria to increase diagnostic specificity [31,32,36,37]. Lung cancer diagnoses were similarly identified using ICD codes (ICD-10 C33–C34 or ICD-9-CM 162/197.0), frequently supplemented by registry confirmation or catastrophic illness certification [32,33,35]. The Chinese case–control study differed methodologically, relying on pathologically confirmed lung cancer cases and documented TB history [38].

The scatter plot in Figure S1 from Supplementary Material S3 illustrates the relationship between the proportion of male participants and the total sample size among the included studies.

Larger nationwide database studies, particularly Moon et al., 2023 [32], and Chai et al., 2022 [36], contributed substantially higher sample sizes compared with the remaining cohorts. In contrast, smaller population-based studies such as Oh et al., 2020 [33], Park et al., 2022 [34], and An et al., 2020 [31], as well as the hospital-based case–control study by Chen et al., 2021 [38], included considerably fewer participants.

Figure S2 from Supplementary Material S3 presents the relationship between the mean or median age and the proportion of male participants in the included studies.

The study populations were predominantly middle-aged to elderly, with the reported ages ranging from 50 years in An et al., 2020 [31] to 67.05 years in Ho et al., 2021 [37]. Most cohorts demonstrated a male predominance exceeding 50%, with the highest male proportion observed in Ho et al., 2021 [37] (72.44%) and the lowest in Chen et al., 2021 [38] (38.16%). Intermediate male proportions were reported in Moon et al., 2023 [32], Park et al., 2022 [34], Chai et al., 2022 [36], and Hong et al., 2024 [35].

### 3.2. Quantitative Synthesis and Meta-Analytic Findings

Table 2 summarizes the adjusted effect estimates evaluating the association between TB and subsequent lung malignancy across the included studies. The majority of studies assessed primary lung cancer risk using hazard ratios (HRs) derived from cohort designs, including An et al., 2020 [31], Moon et al., 2023 [32], Oh et al., 2020 [33], Park et al., 2022 [34], and Chai et al., 2022 [36]. Hong et al., 2024 [35] reported standardized incidence ratios (SIRs) comparing TB patients to the general population, while Ho et al., 2021 [37] evaluated the secondary lung cancer risk. Chen et al., 2021 [38] employed a case–control design and reported the adjusted odds ratios (ORs). Most cohort studies applied multivariable adjustment for major confounders, including age and sex, with several additionally controlling for smoking and comorbidities. The causality reverse was reduced by implementing lag-period exclusions, and studies reporting adjusted HRs for primary lung cancer were considered eligible for the main pooled HR analysis.

Across the studies that were eligible for HR pooling, tuberculosis was consistently associated with an increased risk of subsequent primary lung cancer, with adjusted HRs ranging from 1.23 (1.01–1.49) in the COPD subgroup reported by Park et al., 2022 [34], to 4.18 (3.15–5.56) in An et al., 2020 [31]. Intermediate estimates were observed in Moon et al., 2023 [32], Oh et al., 2020 [33], and Chai et al., 2022 [36], all demonstrating statistically significant associations. Although the effect magnitudes varied, the direction of association remained uniform. Studies excluded from the main HR pooling—such as Hong et al., 2024 [35], Ho et al., 2021 [37], and Chen et al., 2021 [38]—used different effect measures or evaluated different outcomes, but similarly suggested an elevated lung cancer risk following TB.

Table 3 presents the effect estimates converted to the logarithmic scale for meta-analytic pooling. Reported hazard ratios and equivalent ratio measures were transformed into log(HR) values with corresponding standard errors (SE), enabling computation under a random-effects model.

All log-transformed estimates remained positive, indicating a consistent direction of association between tuberculosis and subsequent lung malignancy across studies. Variability in log(HR) and SE values reflects differences in the effect magnitude and study precision, with larger nationwide cohorts demonstrating smaller standard errors and greater statistical weight in the pooled analysis.

**Figure 5:**
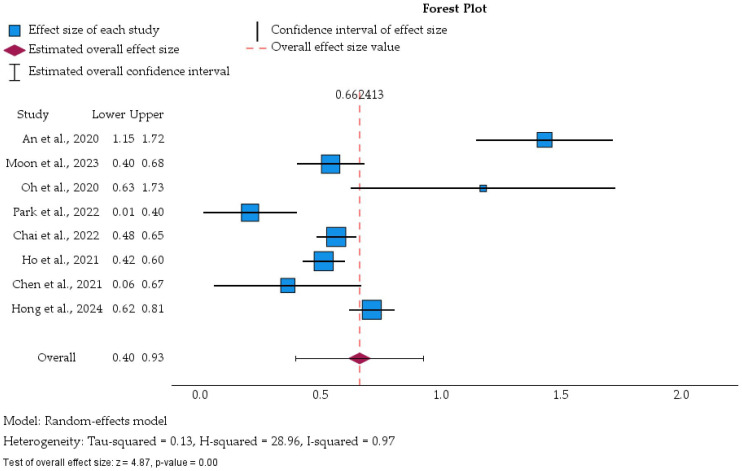
Random-effects forest plot of the association between tuberculosis and subsequent lung malignancy [31,32,33,34,35,36,37,38].

Figure 5 illustrates the pooled effect of prior tuberculosis on subsequent lung malignancy, using a random-effects meta-analysis model [39]. Log-transformed hazard ratios derived from eligible studies [31,32,33,34,35,36,37,38] were synthesized, and individual as well as overall effect estimates are displayed with the corresponding 95% confidence intervals. The statistical model specifications and meta-analytic framework (random-effects REML model, inverse-variance weighting, log-HR transformation) are detailed in Supplementary Material S2 (Tables S2 and S3).

All log-transformed estimates remained positive, indicating a consistent direction of association between tuberculosis and subsequent lung malignancy across studies [31,32,33,34,35,36,37,38]. Although the effect magnitude varied, the pooled estimate demonstrated a statistically significant increased risk under the random-effects model. Larger nationwide cohorts, such as Moon et al., 2023 [32], and Chai et al., 2022 [36], contributed smaller standard errors and consequently greater statistical weight, whereas smaller cohorts and case–control data exhibited wider confidence intervals.

**Figure 6:**
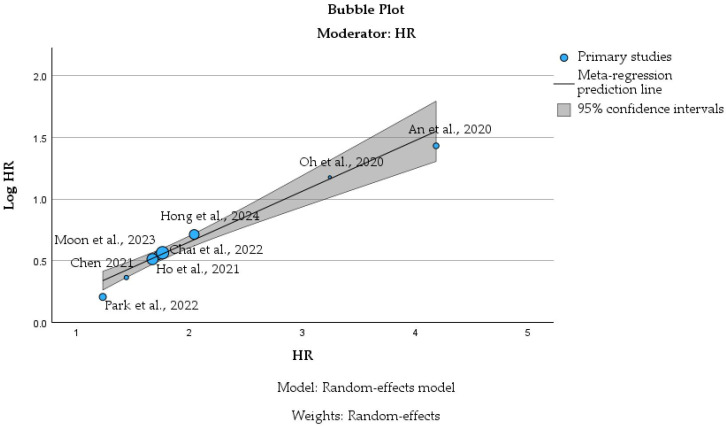
Bubble plot of meta-regression examining hazard ratio as a moderator [31,32,33,34,35,36,37,38].

Figure 6 presents the bubble plot derived from random-effects meta-regression analysis, exploring the relationship between the reported hazard ratios and their log-transformed values across the included studies [31,32,33,34,35,36,37,38]. Bubble size reflects study weight under the random-effects model, while the fitted regression line and shaded 95% confidence region illustrate the meta-regression prediction [40]. The raw data used for this analysis are provided in Table S4 in Supplementary Material S2.

**Figure 7:**
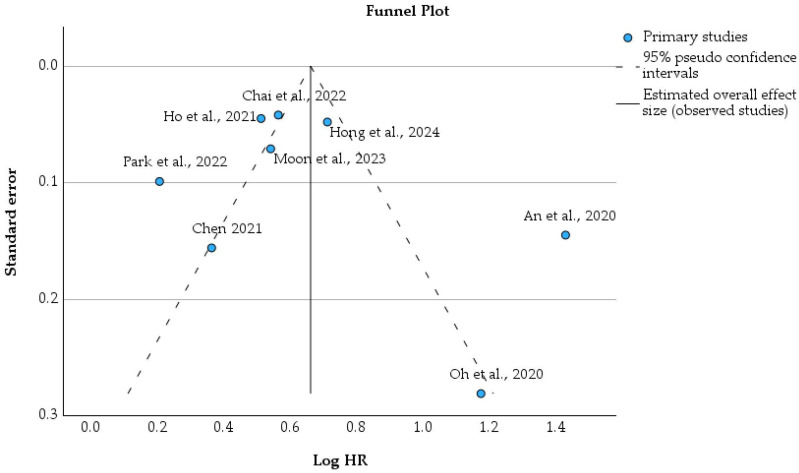
Funnel plot assessing publication bias in the association between TB and subsequent lung malignancy [31,32,33,34,35,36,37,38].

The plot demonstrates a positive linear relationship between HR and log(HR), as expected mathematically, confirming the internal consistency of effect size transformation across studies [31,32,33,34,35,36,37,38]. Larger nationwide cohort studies, including Moon et al., 2023 [32], Chai et al., 2022 [36], and Hong et al., 2024 [35], appear as larger bubbles; thus, they have higher statistical weight, due to smaller standard errors. In contrast, smaller or more heterogeneous cohorts contribute less weight and display wider dispersion around the regression line. Figure 7 illustrates the funnel plot constructed from log-transformed hazard ratios and the corresponding standard errors derived from the included studies [31,32,33,34,35,36,37,38]. The vertical solid line represents the pooled random-effects estimate, while the dashed lines indicate the 95% pseudo-confidence limits [41]. The raw data used for this analysis are also provided in Supplementary Material S3.

Visual inspection of the funnel plot demonstrates moderate asymmetry. Larger nationwide cohort studies with smaller standard errors, including Moon et al., 2023 [32], Chai et al., 2022 [36], and Hong et al., 2024 [35], cluster near the pooled effect size, contributing greater precision. In contrast, smaller or less precise studies, such as Oh et al., 2020 [33] and Chen et al., 2021 [38], appear more dispersed and are located further from the central estimate. Although all studies report positive associations between TB and subsequent lung malignancy, the slight asymmetry suggests potential small-study effects or residual heterogeneity, rather than clear evidence of substantial publication bias [42]. The numerical pooled effect estimate (log HR 0.662; 95% CI 0.396–0.929; *p* < 0.001) and corresponding model parameters are summarized in Supplementary Material S2 (Table S3).

### 3.3. Clinical Case Reports of Tuberculosis-Associated Lung Malignancy

In addition to the population-based cohort studies, six individual case reports (Table S1 from Supplementary Material S3) were identified that described the clinical coexistence or temporal sequence of TB and LC across diverse geographic settings, including Sri Lanka, China, the Philippines, and Korea [43,44,45,46,47,48]. All reported patients were male and ranged in age from 57 to 73 years, reflecting a predominance of older male individuals in whom chronic pulmonary inflammation, structural damage, or active infection may coexist with malignant transformation.

The case reports demonstrate three principal relationship patterns between TB and lung malignancy. First, long-latency post-TB scar carcinoma was described in patients with remote, treated pulmonary TB who developed lung cancer decades later, supporting a chronic inflammation-driven carcinogenic mechanism [43,46]. Second, sequential development of lung cancer shortly after active TB diagnosis was observed, suggesting either inflammatory acceleration of tumorigenesis or potential diagnostic overlap during active infection [44]. Third, several cases presented with concurrent active TB and lung cancer, which was frequently at advanced stages and required combined anti-TB and oncologic therapies [45,47,48]. Histologically, squamous cell carcinoma and adenocarcinoma were the most commonly reported, although rare entities such as pulmonary sarcomatoid carcinoma and small cell lung cancer were also described.

A fully coded numerical representation of all case-level variables used for network construction and graphical modeling is provided in Supplementary Material S2 (Table S1).

**Figure 8:**
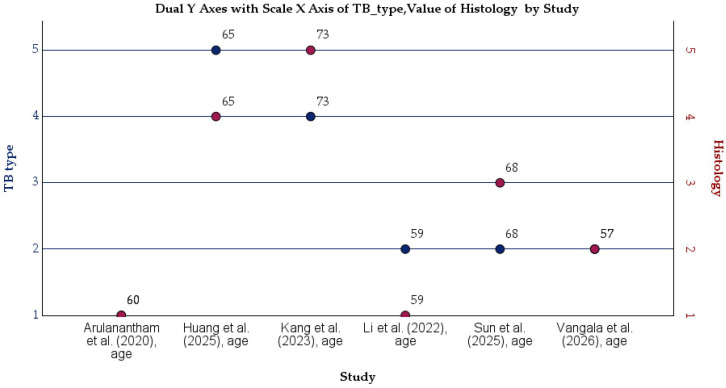
Distribution of TB type and tumor histology across included case reports [43,44,45,46,47,48].

Figure 8 illustrates the distribution of TB type and tumor histology across the six included case reports. The left *y*-axis represents the categorized TB type, while the right *y*-axis denotes the histological subtype of lung malignancy. Individual data points are plotted by study, with age values annotated to provide additional clinical context [49].

The figure highlights the heterogeneity of TB presentations preceding or accompanying lung cancer, including treated/inactive TB, active pulmonary TB, chronic granulomatous TB, and latent TB with reactivation. Histologically, squamous cell carcinoma and adenocarcinoma were the most frequent, although small cell lung cancer and pulmonary sarcomatoid carcinoma were also observed, reflecting diverse oncogenic pathways. No clear age-dependent clustering by TB type or histology is evident.

**Figure 9:**
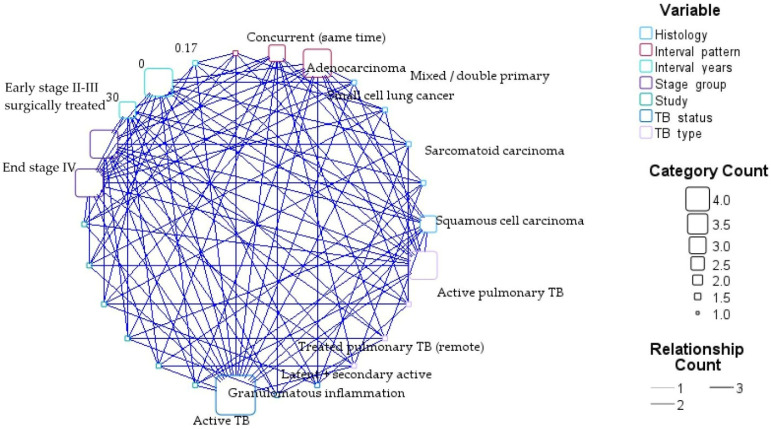
Network relationship map between TB characteristics and LC features.

This relationship map in Figure 9 visualizes the interconnections between TB characteristics (TB type, TB status, and interval pattern), tumor histology, and stage group across the six included cases. The node size reflects the category frequency, while the edge thickness represents the number of observed relationships between variables [50].

The network demonstrates that active TB and concurrent presentation patterns occupy central positions, reflecting frequent co-occurrence with advanced-stage malignancy. Squamous cell carcinoma and adenocarcinoma show multiple connections to both active and prior TB states, supporting heterogeneous mechanistic pathways. Long-latency patterns are primarily linked to post-TB structural lung damage, whereas concurrent cases cluster around advanced disease and systemic therapy.

The quantitative synthesis of population-based cohorts consistently demonstrated a positive association between prior TB and subsequent lung malignancy, with pooled estimates indicating a statistically significant increased risk under a random-effects model. Despite the heterogeneity in study design, lag strategies, and adjustment covariates, the direction of effect remained stable across analyses. Extended pairwise distribution analyses of demographic and clinical variables across the six included case reports are presented in Supplementary Material S2 (Figure S3).

The observed association between tuberculosis and lung cancer must be interpreted in the context of potential confounding, particularly smoking. Although several included studies adjusted for smoking and chronic pulmonary disease, residual confounding cannot be excluded. Additional factors, including socioeconomic status, HIV infection, and environmental exposures, were inconsistently accounted for and may have influenced the reported risk estimates.

### 3.4. Pre-2020 Evidence Supporting Tuberculosis as a Risk Factor for Lung Cancer

Prior to 2020, a substantial body of epidemiological evidence investigated the relationship between TB and subsequent LC risk. These studies, which are predominantly based on large-scale cohort analyses, case–control designs, and meta-analyses, consistently suggested that a history of TB is associated with an increased risk of developing LC. The proposed link has largely been attributed to chronic inflammation, persistent immune activation, and structural alterations of the pulmonary parenchyma following TB infection. Importantly, many of these investigations incorporated national registry data and long-term follow-up, providing valuable insights into temporal associations and risk patterns across diverse populations. A summary of key pre-2020 studies evaluating this association is presented in Table 4.

Pre-2020 evidence consistently demonstrates a positive association between prior TB and an increased risk of LC, as supported by multiple cohort and case–control studies conducted across diverse populations and data sources [51,52,53,54,55,56,57,58]. Large population-based retrospective cohorts from Taiwan reported a substantially increased LC risk among individuals with prior TB, with hazard ratios reaching approximately 3.3, particularly within the first years following TB diagnosis, although the elevated risk persisted during long-term follow-up of up to 10 years [51,52]. Other temporal analyses showed that LC incidence is highest within the first 2–4 years after TB infection, followed by a sustained, albeit attenuated, risk over extended periods [52,58].

Evidence from other geographic regions supports these observations. A cohort study from Hong Kong demonstrated that TB is independently associated with LC mortality, with adjusted hazard ratios confirming its role as a predictor of adverse oncologic outcomes [53]. Similarly, large registry-based studies from Denmark and Finland reported significantly increased LC risk, both shortly after TB diagnosis and beyond five years of follow-up, suggesting both early and long-term carcinogenic effects [57,58]. Case–control studies further corroborate this association, highlighting the interaction between TB history and established risk factors such as smoking, environmental exposure, and familial susceptibility, which may amplify the LC risk in specific subgroups [55,56]. Additionally, broader analyses have suggested associations between TB and multiple malignancies, although these findings raise concerns regarding potential reverse causality [54].

Earlier investigations pre-2020, largely based on administrative databases and registry-linked cohorts, reported elevated LC risk following TB, often with relatively high effect estimates, but were frequently limited by inadequate control of key confounders, particularly smoking, and reliance on heterogeneous diagnostic criteria [51,52,53,54,55,56,57,58]. In contrast, post-2020 studies demonstrate a more refined and methodologically robust approach, incorporating larger and more diverse populations, improved adjustment for confounding variables, including smoking and COPD, and the implementation of lag periods to reduce reverse causality [31,32,33,34,35,36,37,38]. As a result, more recent effect estimates tend to be more conservative (typically aHR ~1.7–2.0), although some studies still report higher risks, particularly in the early period following TB diagnosis [31,32]. Importantly, contemporary data also provide novel insights into subgroup-specific risks, including increased susceptibility among never-smokers and patients with underlying respiratory diseases [34].

## 4. Discussion

Our findings should be interpreted within the context of recent advances in lung cancer management and the complex interaction between malignancy, immunity, and infectious comorbidities such as TB. Major randomized trials and translational studies have demonstrated that immunotherapy and chemoimmunotherapy significantly improve outcomes in NSCLC [59,60,61,62,63,64,65]. Similar to our findings, these studies emphasize the importance of integrating clinical and biological parameters, to refine prognostic stratification and guide therapeutic decision-making.

Smoking remains a major confounding factor in the relationship between TB and lung cancer. Several cohort studies have demonstrated that individuals with a history of TB have an increased risk of lung cancer, even after adjusting for smoking status. Importantly, emerging evidence indicates that this association persists in never-smokers, suggesting that TB itself may act as an independent risk factor. Chronic inflammation, fibrosis, and persistent immune activation are proposed mechanisms underlying this increased susceptibility.

The expanding use of immune checkpoint inhibitors (ICIs) has introduced additional considerations in TB-endemic regions. Nationwide analyses have reported an increased risk of TB reactivation among cancer patients treated with ICIs [66,67]. In line with these observations, our results highlight the importance of careful baseline screening and longitudinal monitoring in patients with overlapping oncologic and infectious risk profiles.

The latency interval between TB infection and subsequent lung cancer development represents a critical dimension of this association. Current evidence indicates that the risk of lung cancer remains persistently elevated for several years following TB diagnosis, with some studies identifying a peak incidence within the first 5–10 years. Several biological mechanisms have been proposed to explain the association between TB and lung cancer, including chronic inflammation, oxidative stress, fibrosis, and immune dysregulation. Persistent inflammatory signaling may promote DNA damage and cellular proliferation, thereby increasing the risk of malignant transformation.

Radiologic differentiation between TB-related lesions and lung malignancy remains challenging. Recent radiomics and deep learning models based on CT imaging have demonstrated improved discrimination between pulmonary tuberculosis granulomas and lung adenocarcinoma [68,69,70]. Similar to our findings, these studies confirm a substantial imaging overlap and support the integration of advanced computational tools to enhance diagnostic precision.

Artificial intelligence applications in high TB-burden settings have further demonstrated improved detection of radiologic patterns that are suggestive of both TB and lung cancer [61], reinforcing our conclusion that multimodal diagnostic approaches are essential.

Finally, immune and hematologic biomarkers have shown prognostic relevance in advanced lung cancer [71], which is consistent with our findings suggesting that immune-related parameters may contribute to risk stratification.

A key aspect that requires clarification is the relatively small number of studies included in the quantitative synthesis. This does not reflect a narrow search strategy or selective inclusion (see Supplementary Material S1), but rather the genuine scarcity of contemporary controlled investigations addressing tuberculosis as a longitudinal risk factor for subsequent lung malignancy. While numerous publications have evaluated lung cancer patients who subsequently develop tuberculosis, particularly in the context of immunotherapy or advanced disease, substantially fewer studies published after 2020 have specifically examined tuberculosis preceding and independently increasing lung cancer risk within controlled cohort designs.

Earlier studies addressing this question exist; however, these have been extensively synthesized in prior meta-analyses and narrative reviews. The present work was intentionally designed to focus on the modern era, characterized by updated diagnostic algorithms, improved TB registries, refined cancer coding systems, and evolving oncologic therapies. The limited number of eligible post-2020 studies therefore highlights a true and clinically relevant research gap. Despite longstanding biological plausibility linking chronic TB-related inflammation to carcinogenesis, contemporary large-scale longitudinal investigations remain insufficient. This gap features the need for prospective, methodologically standardized studies that are specifically designed to evaluate tuberculosis as an independent risk factor for future lung malignancy.

The predominance of studies originating from East Asia represents an important consideration when interpreting the findings of the present analysis. Notably, the marked geographical concentration of available evidence should not be interpreted solely as a limitation of the present analysis, but rather as a reflection of a substantial and clinically relevant gap in the global literature. Despite a comprehensive and systematic search, comparable large-scale, contemporary studies from high-burden regions such as Africa, Eastern Europe, and South America remain strikingly scarce. This imbalance highlights a critical disparity in epidemiological research efforts and raises important concerns regarding the external validity of current risk estimates. Given the heterogeneity in TB epidemiology, host genetic susceptibility, and healthcare access across regions, the absence of data from these populations represents a significant barrier to accurately defining the global relationship between tuberculosis and lung cancer.

This analysis could not be stratified by TB type, treatment status, or lung cancer histology, due to inconsistent reporting across studies. As these factors may influence the association, this represents a limitation and highlights a gap in the available evidence.

## 5. Conclusions

This meta-analysis demonstrates a consistent and statistically significant association between prior tuberculosis and subsequent lung malignancy across contemporary cohort studies. Despite the heterogeneity in study design, lag strategies, and adjustment variables, the direction of effect remained uniform, supporting a robust epidemiological link. The complementary evidence from case reports further illustrates the diverse clinical patterns of interaction, including long-latency scar-associated carcinogenesis and concurrent inflammatory–malignant presentations.

The findings reinforce the biological plausibility that chronic pulmonary inflammation, immune modulation, and structural lung damage may contribute to carcinogenic pathways following TB. At the same time, diagnostic overlap and therapeutic complexity remain important clinical challenges, particularly in TB-endemic settings.

Given the limited number of recent controlled studies, further prospective research with a standardized methodology is warranted. Early surveillance strategies and integrated multidisciplinary approaches may improve risk stratification and outcomes in populations with prior TB exposure.

## Figures and Tables

**Table 1:** Characteristics of cohort studies.

Study	Country	Study Design	Data Source	Sample Size (Total/TB/Non-TB)	Mean/Median Age (Male %)	Follow-up Duration	Lag Period Applied	TB Definition	LC Definition	Key Findings	Major Limitations
An et al., 2020 [31]	South Korea	Retrospective population-based cohort	NHIS–National Sample Cohort	22,656/3776/18,880	58.85% male; majority < 50 yrs	Up to 11 years	No formal exclusion lag; stratified by time	ICD-10 A15–A19 + ≥ 2 anti-TB drugs > 28 days	ICD-10 C33–C34	aHR 4.18 (3.15–5.56); highest risk within 1 year	Possible reverse causality; no histology; administrative coding
Moon et al., 2023 [32]	South Korea	Nationwide retrospective cohort	NHIS database	150,934/75,467/75,467	56.8% male	Median 4.8 yrs	Yes—1-year lag; 2-year sensitivity	ICD-10 A15–A19 + treatment criteria	ICD-10 C33–C34 + V193 code	aHR 1.72 (1.49–1.97); independent of smoking/COPD	Surveillance bias; no TB severity; smoking self-report
Oh et al., 2020 [33]	South Korea	Nationwide cohort	KNHANES linked to cancer registry	20,252/2640/17,612	Mean 62.92 yrs; 42.46% male	Mean 3.9 yrs	Yes—excluded cancer within 6 months	Radiologic inactive TB or self-reported TB	ICD-10 C33–C34 (registry-confirmed)	aHR 3.24 (1.87–5.62); stronger for adenocarcinoma	Short follow-up; small event number; possible misclassification
Park et al., 2022 [34]	South Korea	Nationwide cohort (COPD subgroup)	NHIS–NSC 2.0	13,165 COPD pts (2339 TB/10,826 non-TB)	Mean 66.3 yrs; 52% male	Median 7.7 yrs	Sensitivity excluding 6–12 months	Radiographic history of PTB	ICD-10 C33–C34	sub-HR 1.23 (1.01–1.49); stronger in never-smokers	COPD claims-based; TB defined radiographically
Hong et al., 2024 [35]	South Korea	Population-based cohort	Gyeonggi TB registry + NHIS linkage	35,140 TB patients (no internal control)	57.9% male	Mean 8.0 yrs	Yes—excluded cancer within 1 year	Mandatory TB surveillance registry	ICD-10 C33–C34 + catastrophic illness code	SIR 2.04 (1.85–2.23) vs. general population	No internal control; screening bias; residual confounding
Chai et al., 2022 [36]	Taiwan	Nationwide retrospective cohort	NHIRD	229,225/45,845/183,380	Mean 57.8 yrs; 64% male	Mean 5.8 yrs	Not explicitly stated	ICD-9-CM 010–018	ICD-9-CM 162	aHR 1.76 (1.62–1.91)	No smoking data; administrative coding
Ho et al., 2021 [37]	Taiwan	Nationwide retrospective cohort	NHIRD	20,802/6934/13,868	Mean 67.05 yrs; 72.44% male	Up to 16 yrs	No formal lag	ICD-9-CM 010–011	ICD-9-CM 197.0 (secondary lung cancer)	aHR 1.67 (1.53–1.83) for secondary lung cancer	No smoking data; no staging info; administrative coding
Chen et al., 2021 [38]	China	Case–control	Hospital-based registry	1776 TB/30,763 controls	Median 55 yrs; 38,16% male	N/A (retrospective)		Active or inactive TB history	Pathologically confirmed lung cancer	Adjusted OR 1.44 (1.06–1.95) for lung cancer	No temporal follow-up; hospital-based; recall/coding bias

**Table 2:** Effect estimates of tuberculosis on subsequent lung malignancy.

Study	Country	Outcome Type	Effect Measure	Adjusted Effect Estimate (95% CI)	Adjusted for	Lag Applied	Eligible for Main HR Pooling
An et al., 2020 [31]	Korea	Primary LC	HR	4.18 (3.15–5.56)	Age, sex, income, smoking	No	Yes (sensitivity)
Moon et al., 2023 [32]	Korea	Primary LC	HR	1.72 (1.49–1.97)	Age, sex, BMI, smoking, alcohol, CCI	Yes (1 year; 2-year sensitivity)	Yes
Oh et al., 2020 [33]	Korea	Primary LC	HR	3.24 (1.87–5.62)	Age, sex, smoking, BMI, education	Yes (6-month exclusion)	Yes
Park et al., 2022 [34]	Korea	Primary LC	Sub-HR	1.23 (1.01–1.49)	Age (time scale), sex, smoking, CCI	Sensitivity exclusion	Yes
Hong et al., 2024 [35]	Korea	Primary LC	SIR	2.04 (1.85–2.23)	Age/sex standardized	Yes (1-year)	No (separate analysis)
Chai et al., 2022 [36]	Taiwan	Primary LC	HR	1.76 (1.62–1.91)	Age, sex, comorbidities, income	Not specified	Yes
Ho et al., 2021 [37]	Taiwan	Secondary LC	HR	1.67 (1.53–1.83)	Age, sex, comorbidities	No	No (different outcome)
Chen et al., 2021 [38]	China	Primary LC	OR	1.44 (1.06–1.95)	Age, sex, ethnicity	Not applicable (case–control design; no lag period applied)	No (case–control; OR estimate)

**Table 3:** Effect estimates converted for meta-analysis (log scale).

Study	Country	Outcome Type	HR (95% CI)	log(HR)	SE
An et al., 2020 [31]	Korea	Primary LC	4.18 (3.15–5.56)	1.431	0.145
Moon et al., 2023 [32]	Korea	Primary LC	1.72 (1.49–1.97)	0.542	0.071
Oh et al., 2020 [33]	Korea	Primary LC	3.24 (1.87–5.62)	1.176	0.281
Park et al., 2022 [34]	Korea	Primary LC	1.23 (1.01–1.49)	0.207	0.099
Hong et al., 2024 [35]	Korea	Primary LC	2.04 (1.85–2.23)	0.713	0.048
Chai et al., 2022 [36]	Taiwan	Primary LC	1.76 (1.62–1.91)	0.565	0.042
Ho et al., 2021 [37]	Taiwan	Secondary LC	1.67 (1.53–1.83)	0.513	0.045
Chen et al., 2021 [38]	China	Primary LC	1.44 (1.06–1.95)	0.364	0.156

**Table 4:** Summary of pre-2020 studies evaluating tuberculosis as a preceding risk factor for lung cancer.

Study	Country	Study Design	Data Source	Sample Size (Total/TB/Non-TB)	Mean/Median Age (Male %)	Follow-Up Duration	Lag Period Applied	TB Definition	LC Definition	Key Findings	Major Limitations
Yu et al., 2011 [51]	Taiwan	Retrospective cohort	National Health Insurance Database	716,872/4480 TB/matched controls	Not specified (male predominant)	Up to 10 years	Yes (early cases excluded)	ICD-9 codes for TB	ICD-9 lung cancer diagnosis	TB associated with significantly increased LC risk (HR ~3.3), particularly within first years after infection	Residual confounding (smoking not fully controlled), administrative data
Wu et al., 2011 [52]	Taiwan	Retrospective cohort	Ambulatory care and inpatient discharge records	5657/23,984	58 (41–72); 68.2%	Long-term (≥8 years)	Yes	ICD-based TB diagnosis	ICD-based LC diagnosis	The incidence rate of LC ((269 of 100,000 person-years) was significantly higher in the TB patients than that in the controls (153 of 100,000 person-years) (IRR—1.76). Compared with the controls, the IRRs of LC in the TB cohort were 1.98 at 2 to 4 years, 1.42 at 5 to 7 years, and 1.59 at 8 to 12 years after TB infections	The early symptoms of occult lung cancer could have been diagnosed incorrectly as TB before lung cancer diagnosis. Smoking history was not available. Thus, the authors were unable to adjust for smoking as a contributing factor
Leung et al., 2010 [53]	Hong Kong	Cohort study	TB notification registry from 1993 to 2003	516/60,723	73.2 ± 6.1, 62.4%	~5–10 years	Limited	Microbiologically/clinically confirmed TB	Histologically confirmed LC	TB was associated with death due to LC (RR 2.61), it remained an independent predictor of LC death (aHR 2.01)	No non-TB control group, limited confounder adjustment
Everatt et al., 2017 [54]	Lithuania	Case–control/cohort analysis	Lithuanian Tuberculosis registry (1998–2012)	21,986/	47.1 (12.9); 70.3%	6.2 (4.4) years	Yes	Medical records/claims-based TB	Cancer registry-confirmed LC	TB was associated with leukemia, Hodgkin lymphoma, bone, mesothelial and soft tissue, as well as other cancers	Potential bias due to reverse causality if occult cancer caused a weakening of immunity and malnutrition, resulting in *Mtb* infection or reactivation
Lo et al., 2011 [55]	Taiwan	Case–control	Part of GELAC, molecular epidemiological study on susceptibility markers for LC	288/30	59.54 ± 13.02	Several years	Yes	Demographic characteristics, smoking habit, exposure to environmental tobacco smoke, medical history of lung diseases, family history of LC, and female characteristics were collected from a structured questionnaire	Registry-confirmed	Females exposed to tobacco (OR = 1.39) with a history of TB and with family history of LC in first-degree relatives (OR = 2.44) had higher risk of LC, while subjects with a history of hormone replacement therapy were protective	Confounding (smoking), elderly-only sample
Lim et al., 2011 [56]	China	Case–control	Five major public sector hospitals in Singapore	433/1375	63.0 ± 12.5	Long-term	Yes	Structured questionnaire was administered in person	Registry-confirmed	TB (OR 1.58, 95% CI 0.95– 2.62) appeared to be associated with an increased risk of LC	Only a subset of participants provided blood samples
Simonsen et al., 2014 [57]	Denmark	Cohort	Danish nationwide databases (1978– 2011)	15,024/	Various group ages, 56.1%	Long-term	Yes	Registry-based TB	Cancer registry	Absolute cancer risk 3 months after TB was 1.83% (SIR 11.09); 2.24-fold increased risk beyond 5 years for LC after TB	Residual confounding, surveillance bias
Shiels et al., 2013 [58]	Finland	Cohort	Alpha-Tocopherol, Beta-Carotene cancer prevention study (1985–2005)	185	Various group ages, 100%	Several years	Yes	Clinical/claims-based	Registry-confirmed	TB associated with a two-fold rise in LC risk (HR = 1.97), with significant associations observed for incident (HR = 2.05) and prevalent TB (HR = 1.82). LC risk was greatest in the two-year window after TB diagnosis (HR = 5.01). Only association for SCC was statistically significant	Surveillance bias may occur if people with tuberculosis are more apt to receive medical care and testing that may lead to lung cancer diagnosis

## Data Availability

No new data were generated.

## Supplementary Materials

The following supporting information can be downloaded at: https://www.mdpi.com/article/10.3390/cancers18071097/s1, Supplementary Material S1—PRISMA flow diagram details; Supplementary Material S2—Case-level coding frameworkClinical characteristics and coded variables of tuberculosis-associated lung cancer case reports (Table S1), meta-analytic model specifications (Table S2), pooled effect summary (Table S3), raw effect size data used for meta-analysis calculations (Table S4), and additional pairwise graphical analyses (Figures S1–S3); Supplementary Material S3—Raw effect size data used for meta-analysis calculations. Figure S1: Distribution of sample size according to male proportion across included studies. Figure S2: Age distribution in relation to male proportion across included studies. Figure S3: Pairwise distribution of demographic and clinical characteristics across reported case studies.

## Abbreviations

The following abbreviations are used in this manuscript:

TB	Tuberculosis
LC	Lung cancer
Mtb	*Mycobacterium tuberculosis*
NSCLC	Non-small cell lung cancer
SCLC	Small cell lung cancer
SCC	Squamous cell carcinoma
COPD	Chronic obstructive pulmonary disease
HR	Hazard ratio
sub-HR	Subdistribution hazard ratio
SIR	Standardized incidence ratio
OR	Odds ratio
CI	Confidence interval
SE	Standard error
ICD	International classification of diseases
ICD-10	International classification of diseases, 10th revision
ICD-9-CM	International classification of diseases, 9th revision, clinical modification
NHIS	National health insurance service
NHIS–NSC	National health insurance service–national sample cohort
NHIRD	National health insurance research database
KNHANES	Korea national health and nutrition examination survey
CCI	Charlson comorbidity index
BMI	Body mass index
ICI	Immune checkpoint inhibitor
PD-1	Programmed death-1
PD-L1	Programmed death-ligand 1
PRISMA	Preferred reporting items for systematic reviews and meta-analyses
