# Supplementary material for: Tuberculosis Preceding Lung Cancer: A Contemporary Meta-Analysis Revealing a Critical Gap in Post-2020 Evidence
Source: Cancers (Basel). 2026 Mar 28;18(7):1097. doi: 10.3390/cancers18071097 (PMC13072224; doi:10.3390/cancers18071097)

SUPPLEMENTARY MATERIAL

Table S1: Clinical characteristics and coded variables of tuberculosis-associated lung cancer case reports included in the descriptive synthesis

Study	Country	Age	Sex	TB type	Interval years	Interval pattern	TB Status	Histology	Stage group	Treatment	Outcome
Arulanantham et al. (2020)	Sri Lanka	60	1	1	30	1	2	1	1	6	1
Sun et al. (2025)	China	68	1	2	0.17	2	1	3	2	2	2
Vangala et al. (2026)	Philippines	57	1	2	0	3	1	2	2	3	3
Huang et al. (2025)	China	65	1	5	30	1	1	4	2	5	2
Li et al. (2022)	China	59	1	2	0	3	1	1	1	4	4
Kang et al. (2023)	Korea	73	1	4	0	3	3	5	1	1	1

Coding:

Sex

1 = Male
0 = Female

TB Type

1 = Treated pulmonary TB (remote)
2 = Active pulmonary TB
3 = Latent TB
4 = Chronic granulomatous TB
5 = Latent + secondary active

Interval Pattern

1 = Long-latency (>5 years)
2 = Sequential (active TB → cancer within short time)
3 = Concurrent (same time)

Interval (Years) — Numeric

30
0.17 (≈ 2 months)
0 (concurrent)

TB Status at Cancer Diagnosis

1 = Active TB
2 = Inactive TB
3 = Granulomatous inflammation

Histology Group

1 = Squamous cell carcinoma (SCC)
2 = Adenocarcinoma
3 = Small cell lung cancer (SCLC)
4 = Sarcomatoid carcinoma
5 = Mixed / double primary

Stage Group

1 = Early stage (I–III surgically treated)
2 = Advanced stage (IV)

Treatment Type

1 = Surgery only
2 = Anti-TB + chemotherapy
3 = Anti-TB + systemic therapy
4 = Anti-TB + immunotherapy
5 = Chemotherapy + anti-TB
6 = Surgery + oncology referral

Outcome

1 = Stable
2 = Progressive disease

3 = Advanced disease
4 = Complication (TEN)

Table S2: Meta-analysis summary

Data Type	Pre-calculated
Outcome Type	Continuous
Effect Size Measure	Log HR
Model	Random-effects
Weight	Inverse-variance
Estimation Method	REML
Standard Error Adjustment	None

a. Random-effects weights including both within- and between-study variance.

Table S3: Effect size estimates

	Effect Size	Std. Error	Z	Sig. (2-tailed)	95% Confidence Interval	
					Lower	Upper
Overall	.662	.1361	4.868	<.001	.396	.929

Table S4: Raw effect size data used for meta-analysis calculations

Study	Log HR	SE	Lower CI	Upper CI	HR	Lower HR	Upper HR	Weight	Study ID
An et al., 2020	1.431	0.145	1.15	1.72	4.18	3.15	5.56	1.00	1.00
Moon et al., 2023	0.542	0.071	0.40	0.68	1.72	1.50	1.98	1.00	2.00
Oh et al., 2020	1.176	0.281	0.63	1.73	3.24	1.87	5.62	1.00	3.00
Park et al., 2022	0.207	0.099	0.01	0.40	1.23	1.01	1.49	1.00	4.00

Chai et al.,<br>2022	0.565	0.04<br>2	0.48	0.65	1.7<br>6	1.62	1.91	1.00	5.00
Ho et al., 2021	0.513	0.04<br>5	0.42	0.60	1.6<br>7	1.53	1.82	1.00	6.00
Chen 2021	0.364	0.15<br>6	0.06	0.67	1.4<br>4	1.06	1.95	1.00	7.00
Hong et al.,<br>2024	0.713	0.04<br>8	0.62	0.81	2.0<br>4	1.85	2.23	1.00	8.00

Figure S1: Pairwise scatter plot matrix of sample size, age, and male percentage

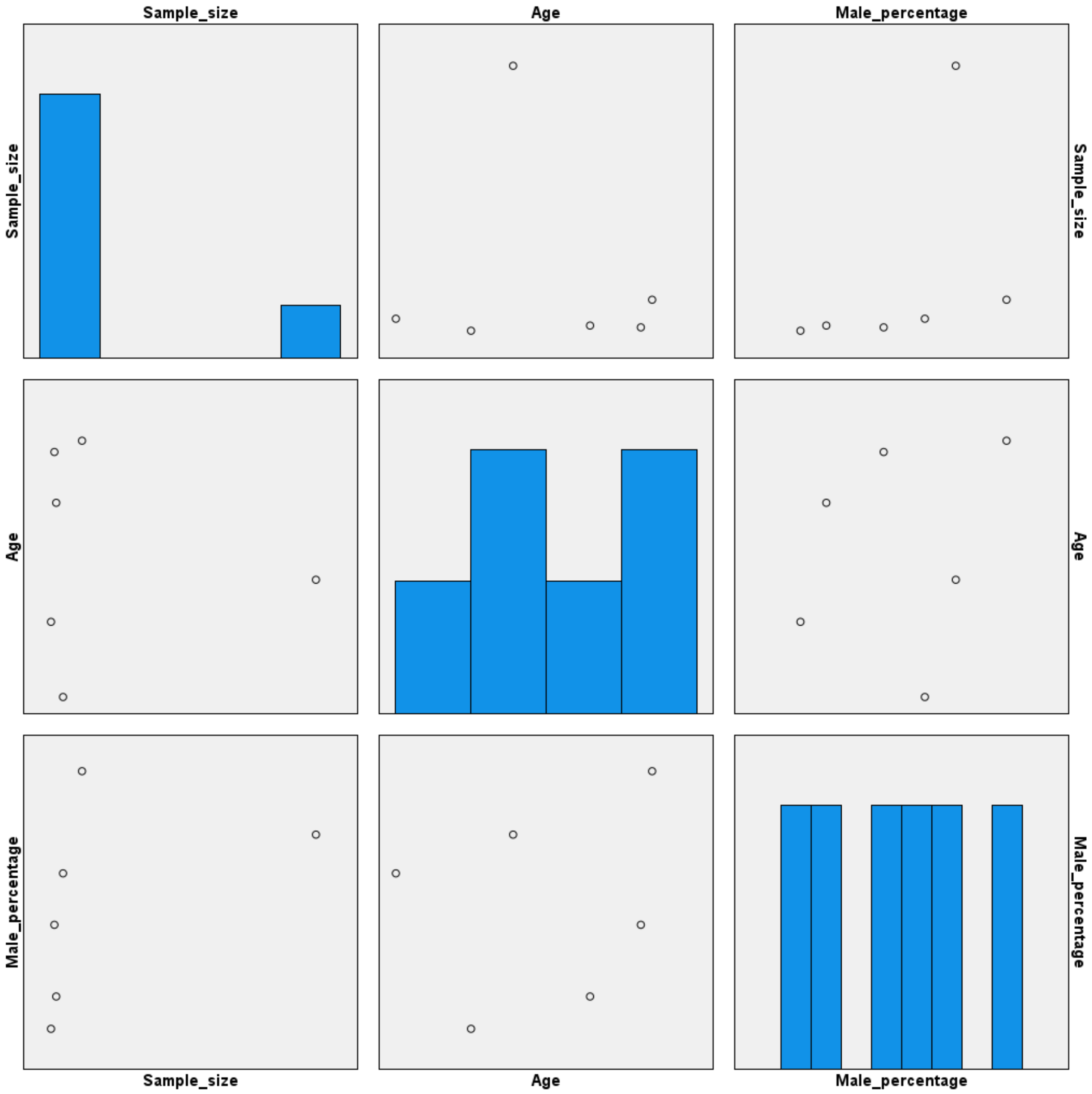

Figure S2: Pairwise relationships between sample size, age, and male percentage across included cohort studies

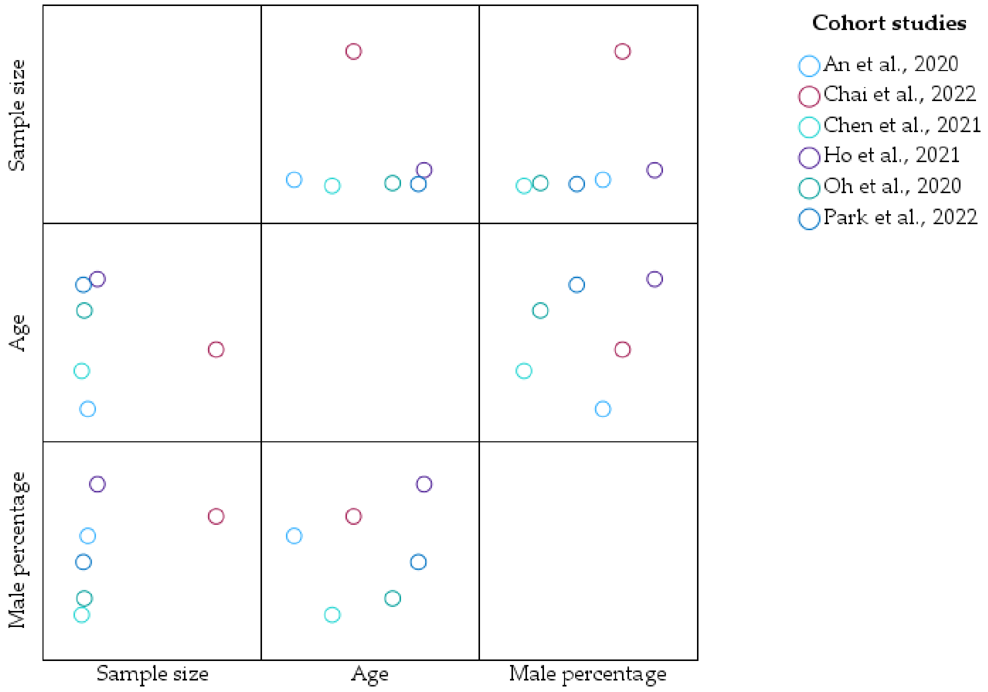

Figure S3: Pairwise distribution of demographic and clinical characteristics across reported case studies

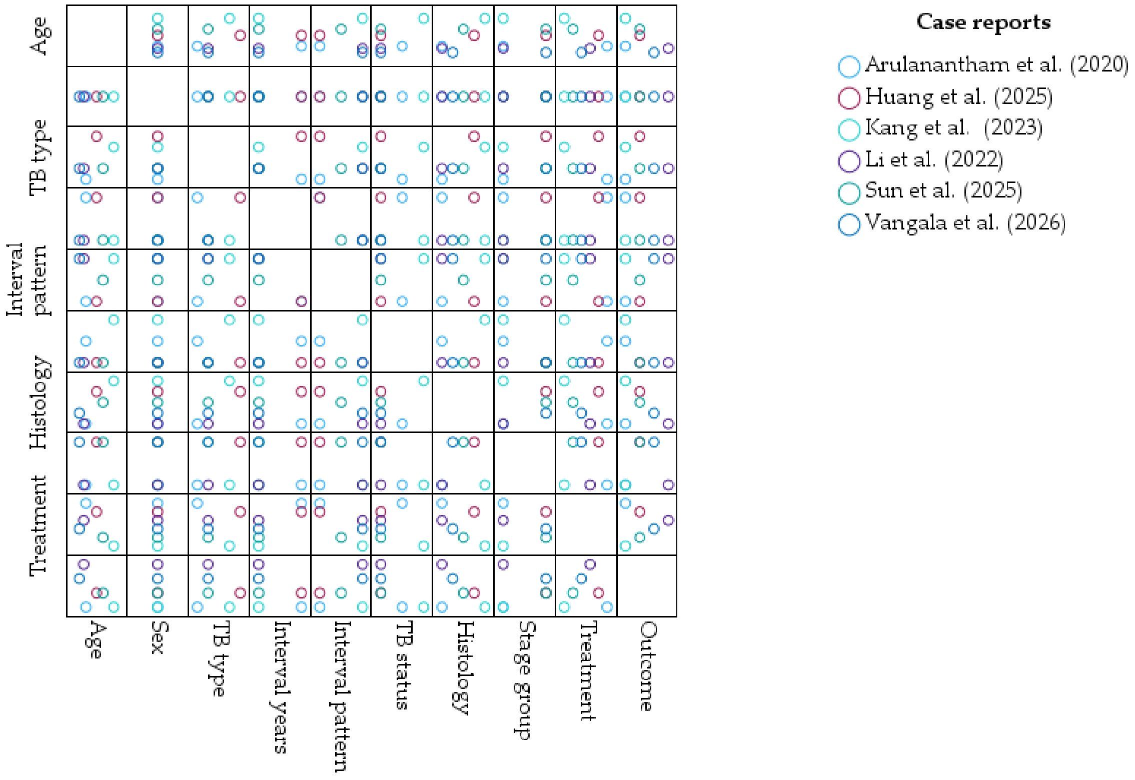

Supplement: Supplementary file 1 [file cancers-18-01097-s001.zip › Supplementary material S2.pdf]
