# Supplementary material for: Tuberculosis Preceding Lung Cancer: A Contemporary Meta-Analysis Revealing a Critical Gap in Post-2020 Evidence
Source: Cancers (Basel). 2026 Mar 28;18(7):1097. doi: 10.3390/cancers18071097 (PMC13072224; doi:10.3390/cancers18071097)
Supplement: Supplementary file 1 [file cancers-18-01097-s001.zip › Supplementary material S3.pdf]

SUPPLEMENTARY MATERIAL

Figure S1. Distribution of sample size according to male proportion across included studies.

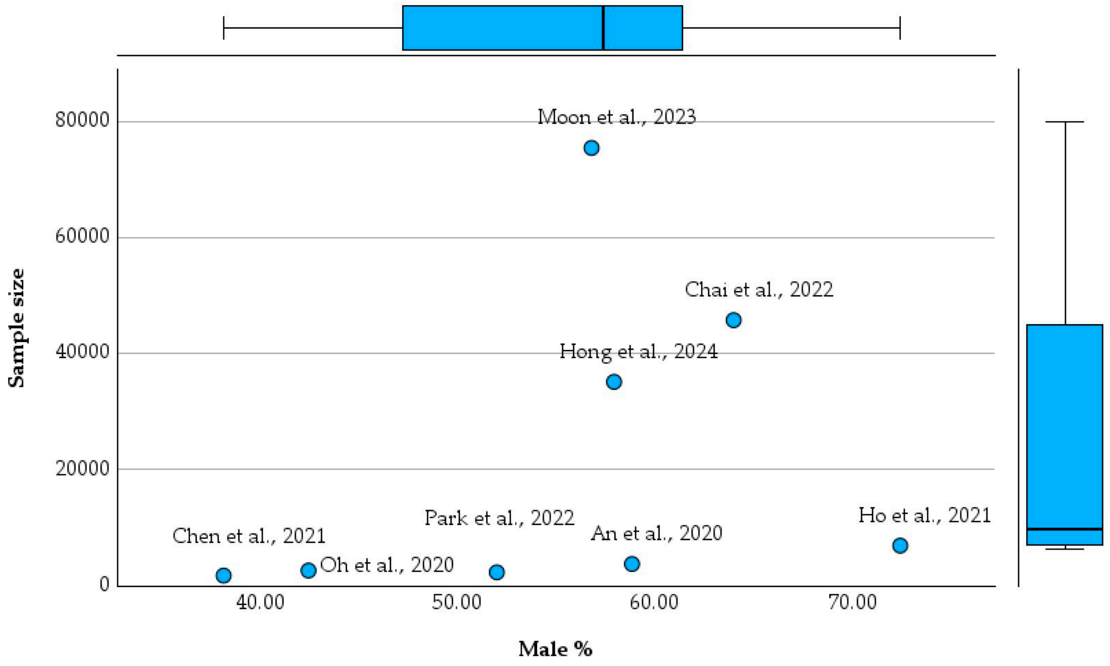

Figure S2. Age distribution in relation to male proportion across included studies.

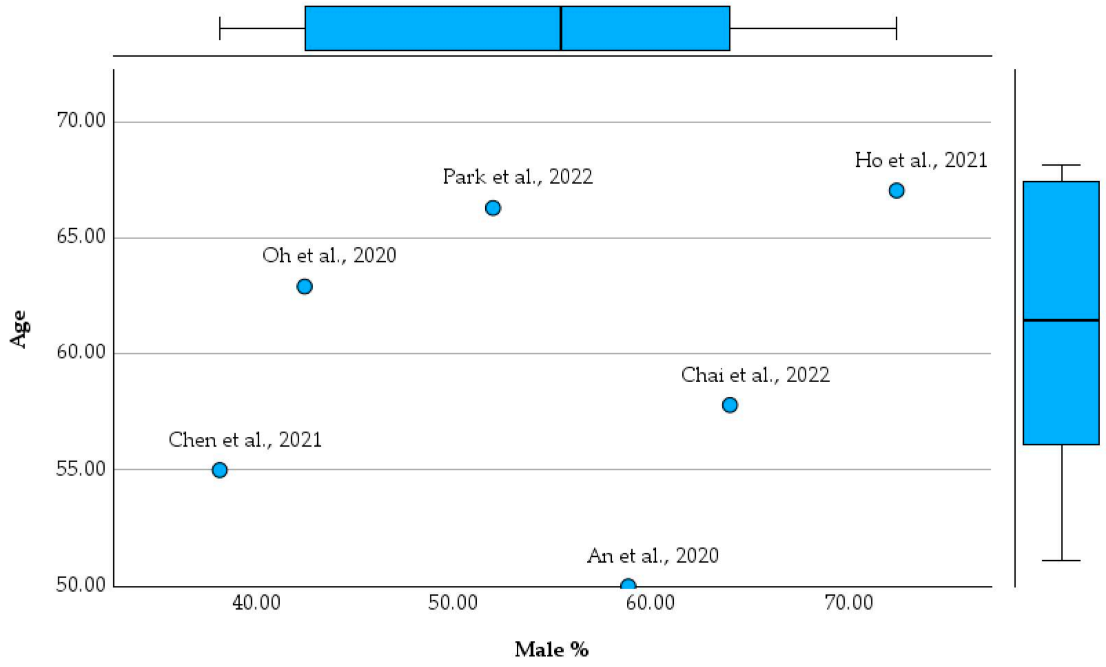

Table S1. Reported cases of TB preceding or present at LC diagnosis.

Study	Country	Age / Sex	TB Type	Interval TB → Cancer	TB status at Cancer diagnosis	Tumor histology	Stage	Treatment	Outcome	Relationship pattern
Arulananth am et al. (2020) [43]	Sri Lanka	60 / M	Treated pulmonary TB	30 years	Inactive; post-TB destroyed lung	Moderately differentiated squamous cell carcinoma	pT4N0Mx	Pneumonect omy	Stable post-op; oncology referral	Long-latency post-TB scar carcinoma
Sun et al. (2025) [44]	China	68 / M	Active pulmonary TB	~2 months	Active TB	SCLC	Stage IV	Anti-TB therapy + chemotherap y	Progressive disease	Sequential active TB → cancer
Vangala et al. (2026) [45]	Philip pines	57 / M	Active pulmonary TB	Concurrent	Active TB (PCR confirmed)	Metastatic lung adenocarcinoma	Stage IV	Anti-TB + systemic therapy	Advanced disease	Concurrent TB + lung cancer
Huang et al. (2025) [46]	China	65 / M	Latent TB (30 yrs prior) + secondary active TB	30 years (latent history)	Active TB at diagnosis	Pulmonary sarcomatoid carcinoma	Stage IVB	Chemothera py + anti-TB	Disease progression	Long-latency + active recurrence
Li et al. (2022) [47]	China	59 / M	Active pulmonary TB	Concurrent	Active TB	SCC	T3N0M0	Anti-TB + neoadjuvant immunother apy	Developed TEN	Concurrent TB + cancer
Kang et al. (2023) [48]	Korea	73 / M	Chronic granuloma tous TB	Concurrent	Granulomatou s inflammation with TB	SCC + adenocarcinoma (double primary)	Early-stage (surgically treated)	Wedge resections	Post-surgical recovery	Concurrent TB + multiple primaries
